# Leukocyte-derived extracellular DNA contributes to abnormal pressure elevation in the extracorporeal circulation circuit

**DOI:** 10.1038/s41598-019-57173-5

**Published:** 2020-01-16

**Authors:** Nozomi Yashima, Takashi Ito, Kenji Kajiyama, Hiroyuki Maeda, Yasuyuki Kakihana, Ikuro Maruyama

**Affiliations:** 1 Department of Emergency and Intensive Care Medicine, Kagoshima University Graduate School of Medical and Dental Sciences, Kagoshima, Japan; 2 Department of Systems Biology in Thromboregulation, Kagoshima University Graduate School of Medical and Dental Sciences, Kagoshima, Japan; 3 Department of Anesthesiology, Yamagata University Graduate School of Medical Science, Yamagata, Japan; 4 Cardiovascular Device Team, Development Department, Surgical & Therapeutical Business Unit, JMS Co., Ltd., Hiroshima, Japan

**Keywords:** Mechanisms of disease, Cardiac device therapy

## Abstract

An abnormal elevation in pressure is a serious complication involving the extracorporeal circulation circuit. Clot formation might be associated with this complication, but the precise mechanism of an abnormal elevation in pressure has not been identified. We investigated sufficient conditions for in-circuit elevation in pressure using an *ex vivo* re-circulation circuit with porcine blood. Specifically, we investigated the effect of blood conditions, the type of anticoagulation, and pro-inflammatory stimulation on in-circuit pressure. We also examined the cause of an abnormal elevation of in-circuit pressure by specifically degrading DNA, RNA, or protein components of an obstructed filter and by using immunofluorescent techniques. Neither a change in temperature nor change in pH in the blood increased in-circuit pressure. In contrast, long-term storage of blood, pro-inflammatory stimulation by phorbol myristate acetate, and heparin administration significantly increased in-circuit pressure. Abnormal in-circuit elevation in pressure was associated with deposition of extracellular DNA on the outlet surface of the filter. Administration of DNase resulted in a rapid decline of in-circuit pressure. In an *ex vivo* re-circulation circuit system, extracellular DNA deposition on the filter is responsible for an abnormal in-circuit elevation in pressure. Senescent leukocytes, stimulated leukocytes, and heparin exposure are associated with extracellular DNA deposition.

## Introduction

Cardiopulmonary bypass (CPB) is important in maintaining the circulation and respiration during cardiac surgery. Abnormal elevation in pressure is a serious complication of CPB and has been reported in 0.03–4.3% cases of CPB^1–9^. This abnormal elevation in pressure has been termed abnormal inlet pressure elevation^1^, abnormal pressure gradient^2^, high-pressure excursion^3^, increased resistance in the oxygenator^4^, oxygenator failure^5,6^, increased internal pressure levels^7^, and oxygenator thrombosis^8^.

Abnormal elevation in pressure may occur at three narrowed sections in CPB equipment, including the venous reservoir, oxygenator, and arterial line filter. Previous studies have suggested risk factors for an abnormal elevation in pressure in CPB, such as older age^2^, male sex^1,3^, coronary artery disease^1–3,10^, and polycythemia vera^11^. Hypothermia and alkalemia might also be associated with an abnormal elevation in pressure, although these factors are not sufficient by themselves^3,9^. Fibrin deposition inside CPB equipment might be involved in the underlying mechanism^1,2,6,9,10,12^. However, whether intensification of anticoagulation can ameliorate an abnormal elevation in pressure is unclear because most patients have already received a large amount of unfractionated heparin^1,2,11,12^. Therefore, the precise mechanism of an abnormal elevation in pressure during CPB is unknown.

To determine the underlying mechanisms of an abnormal elevation in pressure during CPB, we developed an extracorporeal recirculation circuit in which in-circuit pressure could be monitored and a filter could be easily removed and analyzed. In this *ex vivo* circuit, we used a removable filter with a pore size of 40 µm because the pore sizes of a cardiotomy filter and arterial line filter in CPB equipment are approximately 40 µm, while the span of oxygenator fibers is approximately 50–250 µm. Using the *ex vivo* circuit system and porcine blood, we found that abnormal elevation of in-circuit pressure was associated with leukocyte activation or senescence in this study. Furthermore, we found that extracellular DNA deposition on the outlet of the filter was responsible for the abnormal in-circuit elevation in pressure.

## Results

### Long-term storage of heparinized blood increases in-circuit pressure

**Figure 1:**
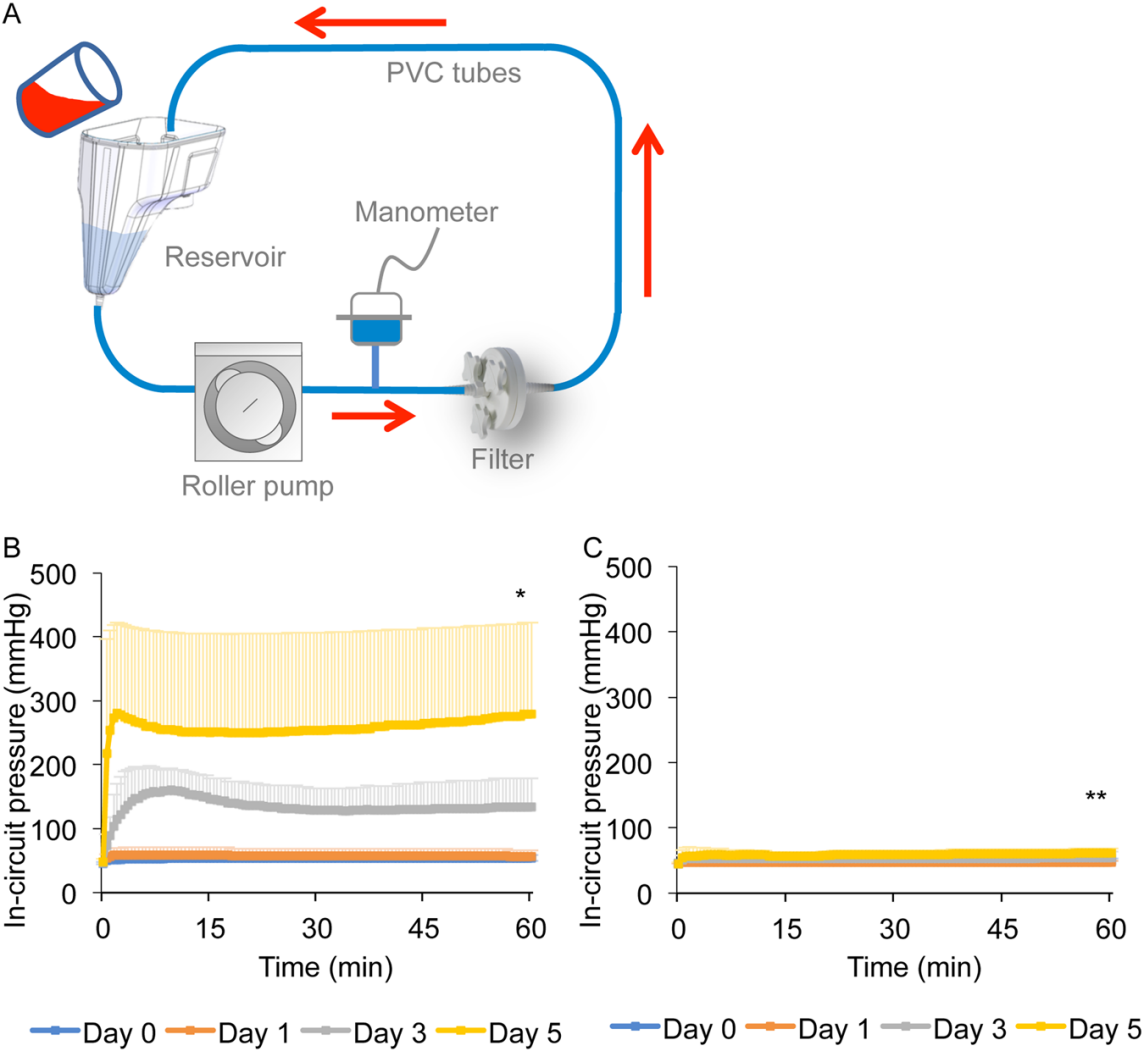
Long-term storage of heparinized blood causes elevation of in-circuit pressure. (**A**) A schema of the extracorporeal recirculation circuit is shown. The circuit consists of polyvinyl chloride (PVC) tubes, a pooling reservoir, a roller pump, a manometer, and a removable filter with a pore size of 40 µm. (**B**) Heparinized blood stored for 0, 1, 3, or 5 days was administered to the circuit and in-circuit pressure was monitored for up to 60 minutes. N = 5 per group. (**C**) Citrated blood stored for 0, 1, 3, or 5 days was administered into the circuit and in-circuit pressure was monitored for up to 60 minutes. N = 5 per group. Repeated measures analysis of variance models were used to analyze changes in the area under the curve (AUC) over time. *p < 0.05, **p < 0.01.

Using the *ex vivo* circuit system (Fig. 1A) and porcine blood, we investigated sufficient conditions for an elevation of in-circuit pressure. In control conditions where porcine blood was freshly collected (day 0) and unstimulated, in-circuit pressure was not increased. Neither a change in temperature ranging from 37 °C to 10 °C nor a change in pH ranging from 8.0 to 6.8 in the circuit system increased in-circuit pressure (data not shown). In contrast, long-term storage of blood significantly increased in-circuit pressure (p < 0.05) if blood was anticoagulated with heparin (Fig. 1B), while in-circuit pressure elevation was minimal if blood was anticoagulated with citrate (Fig. 1C). These findings suggest that the storage term of blood and the type of anticoagulation may be associated with an abnormal elevation in pressure.

### Heparin administration in stored blood leads to extracellular DNA deposition on the outlet of the filter

**Figure 2:**
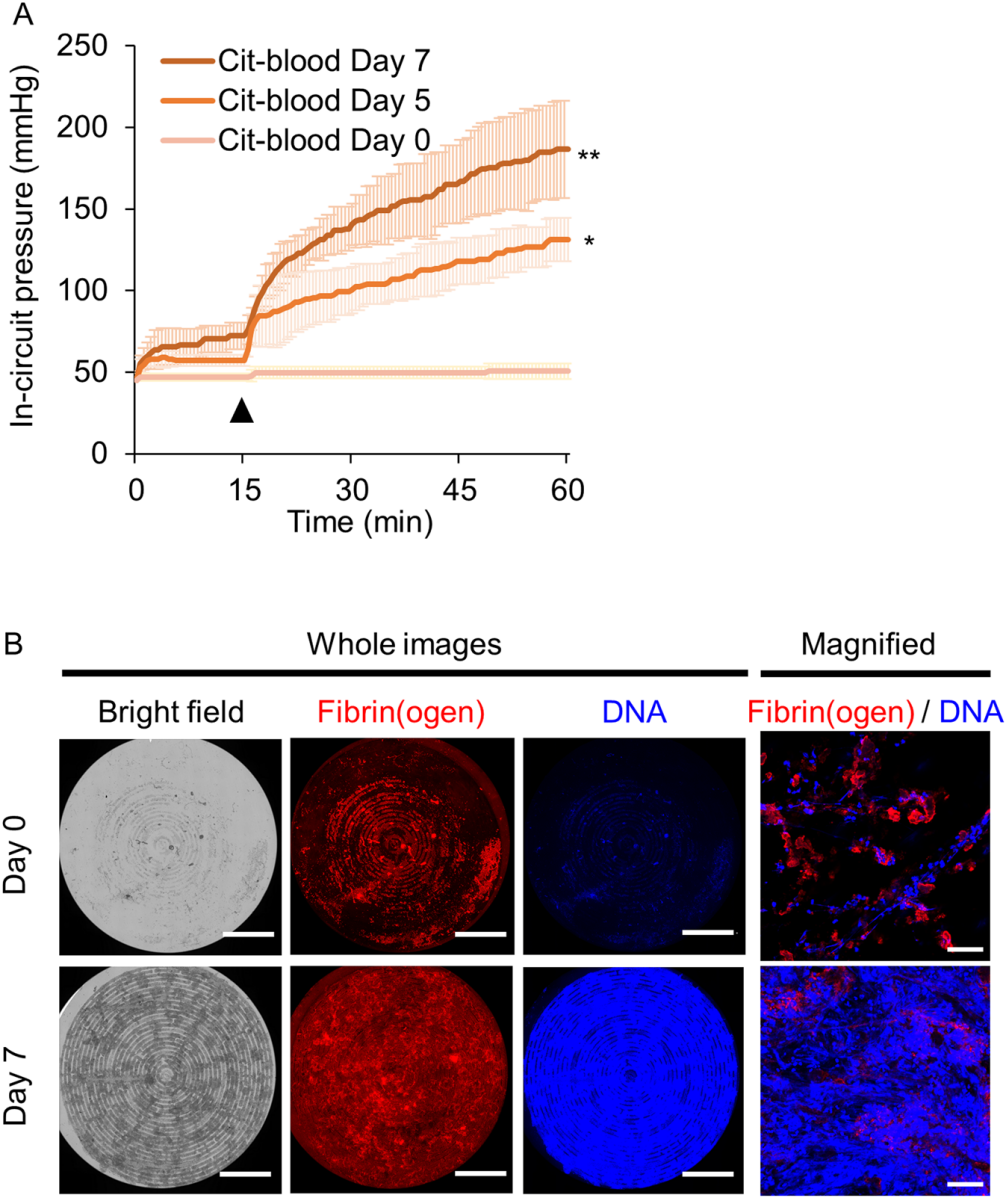
Heparin administration in stored blood leads to extracellular DNA deposition on the outlet of the filter. (**A**) Citrated blood (Cit-blood) stored for 0, 5, or 7 days was administered to the circuit and then 5 U/mL of unfractionated heparin was administered at 15 minutes. In-circuit pressure was monitored for 60 minutes. N = 4 per group. The paired t-test was used to analyze the difference between in-circuit pressure at 15 and 60 minutes. *p < 0.05, **p < 0.01. (**B**) The filter was removed at 60 minutes and analyzed by immunofluorescence. Fibrin(ogen) was labeled in red and DNA was labeled in blue. Whole images of the filters (scale bars: 10 mm) were analyzed by BZ-X700 microscopy and magnified images (scale bars: 50 μm) were analyzed by LSM 700 confocal microscopy. Representative images of N = 5 are shown.

We then investigated whether heparin administration in citrated blood leads to elevation of in-circuit pressure. Heparin administration did not increase in-circuit pressure (pre- vs post-heparin administration, p = 0.092) if blood was fresh (day 0) (Fig. 2A). In contrast, heparin administration significantly increased in-circuit pressure (pre- vs post-heparin administration, p < 0.01) if blood was stored for 5 days or longer. When day 7 stored blood passed through the filter, a grossly visible sticky substance was adhered (Video S1). Immunofluorescent analysis showed that this sticky substance was composed of extracellular DNA and fibrin(ogen) (Fig. 2B). In contrast, little extracellular DNA was observed when day 0 blood was passed through the filter, and some intact nuclei and fibrin(ogen) were observed (Fig. 2B). A three-dimensional reconstitution technique showed that the extracellular DNA layer was located immediately downstream of the filter (Video S2 and Fig. S2). The fibrin(ogen) layer was located downstream of the DNA layer (Fig. S2). These results suggest that DNA is primarily responsible for obstruction of the circuit.

### Leukocytes play a role in elevation of in-circuit pressure

**Figure 3:**
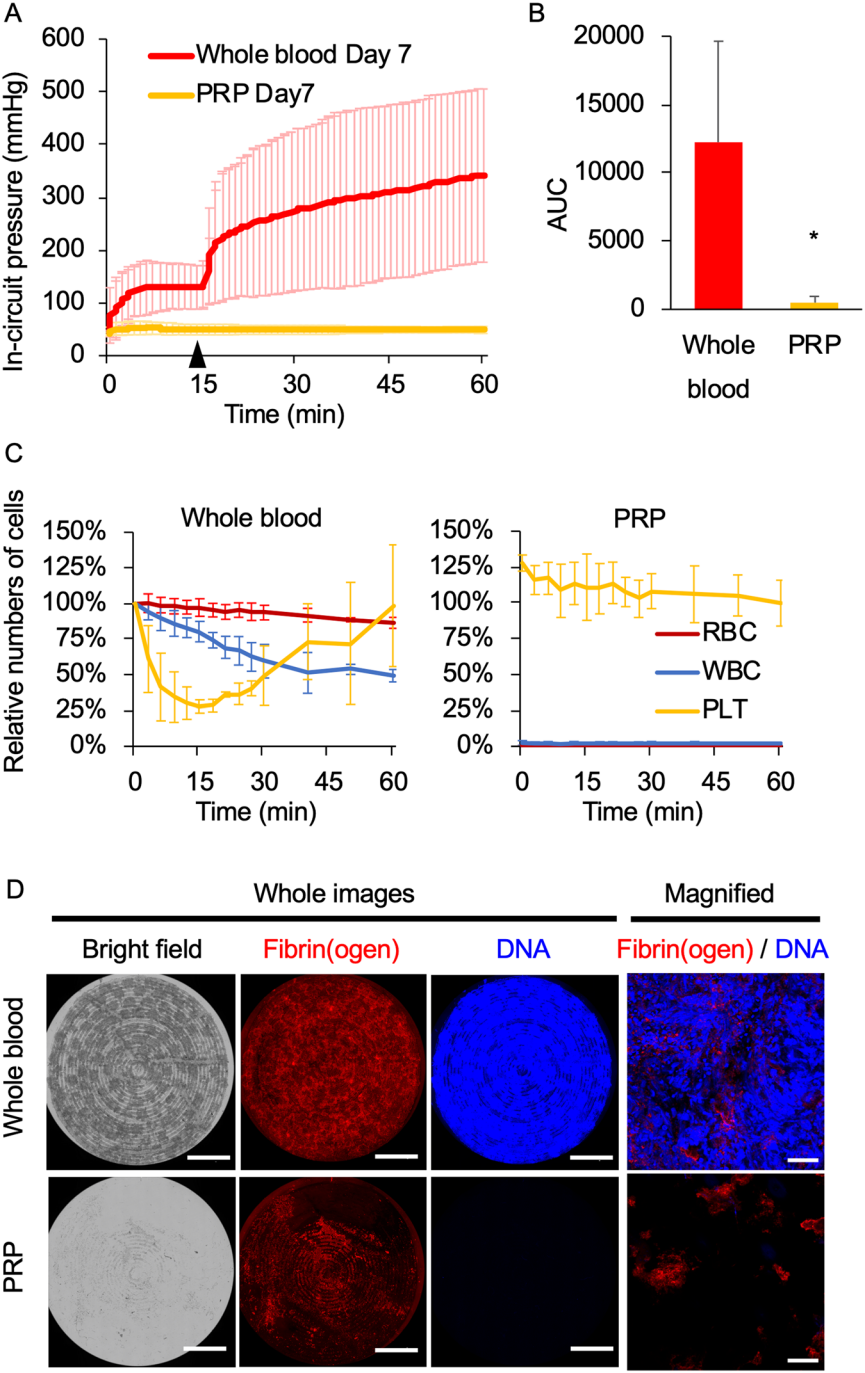
Leukocytes play a role in elevation of in-circuit pressure. (**A**) Whole blood or PRP stored for 7 days was administered to the circuit and then 5 U/mL of unfractionated heparin was administered at 15 minutes. In-circuit pressure was monitored for 60 minutes. N = 6 per group. (**B**) The paired t-test was used to analyze the difference in the area under the curve (AUC) between whole blood and PRP. N = 6 per group. *p < 0.05. (**C**) Relative numbers of erythrocytes (RBC), leukocytes (WBC), and platelets (PLT) to the initial numbers in whole blood are shown. N = 3 per group. (**D**) The filter was removed at 60 minutes and analyzed by immunofluorescence. Fibrin(ogen) was labeled in red and DNA was labeled in blue. Representative images of the whole filter (scale bars: 10 mm) and the magnified view (scale bars: 50 μm) are shown.

We examined whether leukocytes, the dominant nucleated cells in blood, contribute to elevation of in-circuit pressure. We prepared platelet-rich plasma (PRP) in which erythrocytes and leukocytes were almost absent, but platelets were abundant. On day 7, in-circuit pressure was increased if heparin was administered in whole blood (Fig. 3A,B). In contrast, in-circuit pressure was not increased if heparin was administered in PRP (Fig. 3A,B). Decreases of platelets and leukocytes were evident before and after administration of heparin, respectively, in whole blood while they were less obvious in PRP (Fig. 3C). Hemolysis was accompanied by the elevation of in-circuit pressure (Fig. S3). In immunofluorescent analysis, extracellular DNA was detected all over the filter through which whole blood passed, whereas extracellular DNA deposition was not observed on the filter through which PRP passed (Fig. 3D). These findings suggest that platelets and plasma, two important components in thrombus formation, are not sufficient for abnormal elevation in pressure, and leukocytes and/or erythrocytes play an essential role.

### Extracellular DNA contributes to elevation of in-circuit pressure

**Figure 4:**
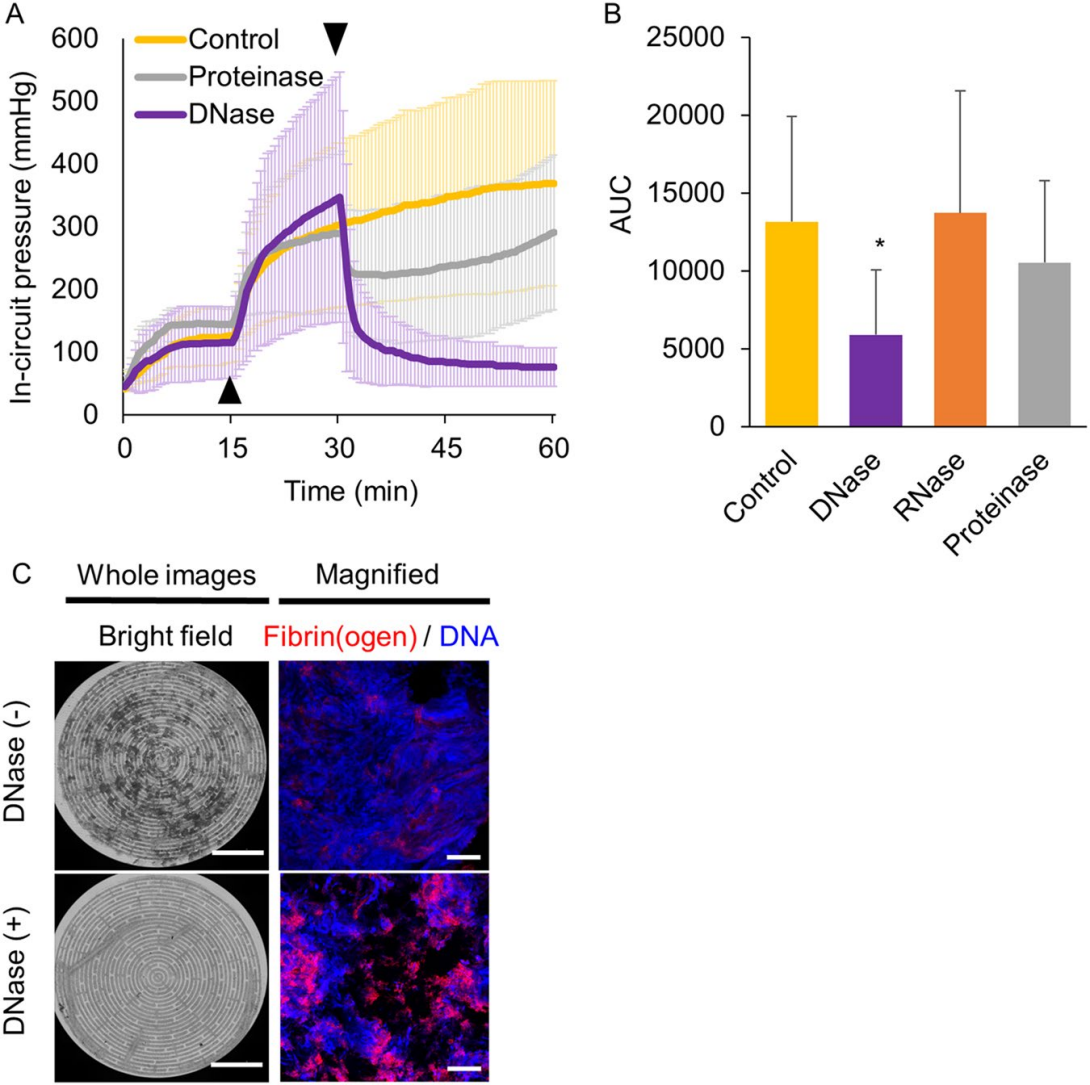
Extracellular DNA contributes to elevation of in-circuit pressure. (**A**) Citrated whole blood stored for 7 days was administered to the circuit and then 5 U/mL of unfractionated heparin was administered at 15 minutes. Deoxyribonuclease (DNase), ribonuclease (RNase), or proteinase was administered at 30 minutes and in-circuit pressure was monitored for up to 60 minutes. N = 4 per group. A waveform of in-circuit pressure in RNase group did not represent because it was almost same as that of no treatment. (**B**) The paired t-test was used to analyze the difference in the area under the curve (AUC) between DNase treatment and no treatment. *p < 0.05. (**C**) The filter was removed at 60 minutes and analyzed by immunofluorescence. Fibrin(ogen) was labeled in red and DNA was labeled in blue. Representative images of the whole filter (scale bars: 10 mm) and the magnified view (scale bars: 50 μm) are shown.

We next examined whether extracellular DNA directly contributes to elevation of in-circuit pressure. We administered DNase into the partially obstructed circuit and monitored in-circuit pressure. DNase treatment immediately decreased in-circuit pressure to a baseline level at 60 minutes (75.9 ± 31.0 mmHg with DNase treatment vs 369.4 ± 163.3 mmHg with no treatment, p < 0.05) (Fig. 4A). In contrast, RNase treatment did not decrease in-circuit pressure (Fig. 4B). Proteinase treatment partially decreased in-circuit pressure. Immunofluorescent analysis showed that extracellular DNA on the surface of the filter was degraded by DNase treatment (Fig. 4C). To determine whether these events were specific to the removable filter used in this study or generally applicable to clinical conditions, we performed the same experiment using the clinically approved venous reservoir, oxygenator, and arterial line filter. In-circuit pressure was elevated at the oxygenator if heparin was administered in whole blood that was stored for 7 days (Fig. S1B). Furthermore, DNase treatment immediately decreased in-circuit pressure. These findings indicate that extracellular DNA is directly responsible for obstruction of the circuit.

We also examined the possibility that endogenous DNase protects the filter from DNA obstruction. We used EDTA, which can inhibit DNase activity by chelating divalent metal cations. Inhibition of endogenous DNase by EDTA significantly increased in-circuit pressure if heparinized blood stored for 3 days was used (EDTA positive vs negative: 465.0 ± 120.0 mmHg vs 226.9 ± 179.8 mmHg at 60 minutes, p < 0.05) (Fig. S4). In contrast, this effect was not observed if heparinized blood on day 0 was used (EDTA positive vs negative: 57.2 ± 4.7 mmHg vs 55.3 ± 5.6 mmHg at 60 minutes). These results suggest that EDTA by itself does not induce obstruction of the filter, but it can exacerbate extracellular DNA-mediated obstruction of the filter.

### Leukocyte stimulation leads to extracellular DNA deposition and elevation of in-circuit pressure

**Figure 5:**
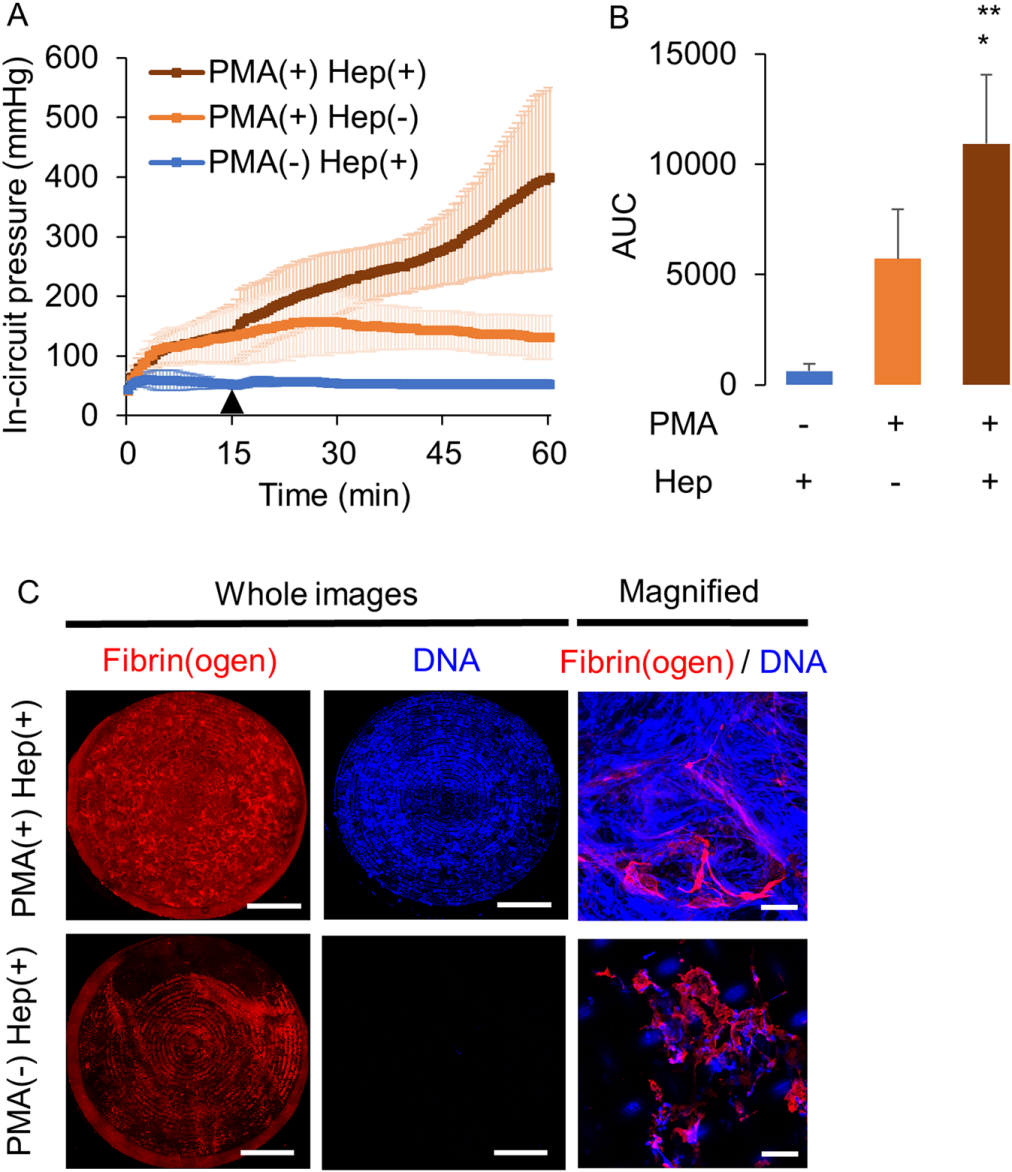
Leukocyte stimulation leads to extracellular DNA deposition and elevation of in-circuit pressure. (**A**) Citrated whole blood on day 0 was stimulated with 0.6 µg/ml phorbol 12-miristate 13-acetate (PMA) at 37 °C for 120 minutes before administration to the circuit. Unfractionated heparin (Hep) was administered at 15 minutes and in-circuit pressure was monitored for up to 60 minutes. N = 4 per group. (**B**) The paired t-test was used to analyze the difference in the area under the curve (AUC) between the PMA-stimulated group and the non-stimulated group. *p < 0.05. (**D**) The filter was removed at 60 minutes and analyzed by immunofluorescence. Fibrin(ogen) was labeled in red and DNA was labeled in blue. Representative images of the whole filter (scale bars: 10 mm) and the magnified view (scale bars: 50 μm) are shown.

Finally, we examined whether in-circuit pressure could be elevated even if blood was fresh. To this end, we used fresh blood and monitored in-circuit pressure under two hypothetical circumstances: transfusion of old blood and inflammatory stimulation. Heparin administration into the mixture of fresh blood, saline, and day 7 stored blood did not increase in-circuit pressure significantly (Fig. S5A). However, immunofluorescent analysis showed deposition of extracellular DNA on the filter surface (Fig. S5B). These findings suggest that transfusion of old blood is not sufficient for elevation of in-circuit pressure by itself but may become the genesis of pore-clogging. Then, we examined whether leukocyte stimulation results in elevation of in-circuit pressure since previous studies have suggested that stimulated leukocytes are prone to releasing DNA into the extracellular space^13^. In-circuit pressure was increased if whole blood on day 0 was stimulated with phorbol 12-miristate 13-acetate (PMA) (Fig. 5A). Heparin administration in PMA-stimulated blood led to a significant further increase in in-circuit pressure (Fig. 5A,B). Immunofluorescent analysis showed that PMA stimulation induced deposition of extracellular DNA all over the filter surface (Fig. 5C). These findings suggest that not only leukocyte senescence, but also leukocyte stimulation, promote elevation of in-circuit pressure.

## Discussion

Our study showed that leukocyte-derived extracellular DNA induced an elevation of in-circuit pressure. Furthermore, heparin injection caused leukocytes to release extracellular DNA. Previous studies have reported an abnormal elevation in pressure in CPB^1–9^. However, no studies have suggested that extracellular DNA can contribute to abnormal elevation in pressure. Extracellular release of DNA from neutrophils, called neutrophil extracellular traps (NETs), was first described in infectious diseases^14^, and the process was named NETosis. This process is morphologically and functionally distinct from other forms of cell death^13,15^. Recently, subsequent studies established that other immune cells also release extracellular DNA (renamed ETosis) in eosinophils^16,17^, basophils^18,19^, monocytes^20^, macrophages^21^, and lymphocytes^22^. Although ETosis was induced by different stimuli for several minutes to hours in these studies, a unique study showed that high hemodynamic forces trigger rapid NET release within 2 minutes^23^. Our data are in good agreement with this previous study. We found that leukocytes, including neutrophils, were disrupted when they passed through the narrow site in the circuit, and extracellular DNA was adhered downstream of the filter.

The elevation of pressure in CPB has unique characteristics, including transient elevation of pressure in some cases^1,2,9^. In some experiments in our study, the same phenomenon was observed, especially using heparinized blood, but not citrated blood. This finding can be explained by endogenous DNase activity. Extracellular DNA is degraded by endogenous DNase, leading to a gradual reduction in elevated in-circuit pressure. Because this enzyme requires a divalent cation to activate, in-circuit pressure is not reduced in blood that is anticoagulated with citrate sodium.

Our results suggest that heparin is also associated with elevation of in-circuit pressure. Heparin is the most common anticoagulant in cardiac surgery because its effects can be measured by monitoring the activated clotting time and reversed by protamine. With regard to the dose of heparin used in this study, 5 U/mL corresponds approximately to 400 U/kg and is thus reasonable in CPB settings^24^. However, some reports have suggested that heparin itself affects functions of leukocytes, besides its role in anticoagulation. Lazarowski *et al*.^25^ reported that heparin alone, ranging from 0.3 to 15 U/mL, could induce *in vitro* aggregation of human polymorphonuclear neutrophils (PMNs). Cairo *et al*.^26^ reported that PMNs stimulated with N-formylmethionyl-leucyl-phenylalanine were aggregated by heparin, although heparin itself did not promote aggregation at low doses. This finding indicates that even low heparin concentrations can promote aggregation of PMNs if they are activated. They considered that this aggregation might be induced by heparin’s highly negative charge and ability to locate the surface of the cell membrane. Gebska *et al*.^27^ showed that apoptotic and necrotic leukocytes had a high affinity for heparin compared with healthy live leukocytes. In our study, aggregation of leukocytes and platelets was observed at day 3 in heparinized blood, but not in citrated blood, in Giemsa-stained thin blood smears (Fig. S6). Some of the aggregates were more than 40 µm. This aggregation of leukocytes led to difficulty for them passing through the filter, leading to physical stress and they were easily destroyed. Accordingly, heparinized blood and blood after adding heparin are likely to raise in-circuit pressure. Similarly, leukocytes stimulated with PMA were aggregated with nuclear degeneration and cytoplasmic vacuolization (Fig. S6).

A reasonable treatment of an abnormal elevation in pressure is to add DNase to the circuit. In our study, DNase treatment resulted in a rapid decrease of raised in-circuit pressure. A previous study created a mouse model of ventilator-induced lung injury^28^ and transfusion-related acute lung injury^29^, which were associated with NET formation in the lungs. This previous study showed that inhalational or intratracheal DNase treatment eliminated NETs and improved arterial oxygen saturation in ventilator-induced lung injury. However, many DNA fragments enter the patient’s body if DNase is added to the oxygenator when an abnormal elevation in pressure occurs. DNA fragments, which is one of the damage-associated molecular patterns, may induce an inflammatory response^30^. Therefore, overall effects of DNase treatment should be investigated in an *in vivo* animal model.

A new oxygenator needs to be developed, which can prevent blood from excessive shear stress, as well as coagulation activity. Our study suggested that extracellular DNA from disrupted leukocytes contributed to elevation of in-circuit pressure. Not only taking account of intra-oxygenator thrombosis but also taking a proper care of leukocyte disruption may be important.

Another strategy to suppress this phenomenon is optimization of anticoagulation therapy. As mentioned above, heparin affects leukocytes, as well as coagulation/anticoagulation factors. We consider that an excessive dose of heparin can be adverse and an optimal dose of heparin should be determined.

Our study has some limitations. First, we showed that leukocyte-derived extracellular DNA contributed to an abnormal elevation in pressure in an *ex vivo* circuit, but it may be merely a partial explanation of abnormal elevation in pressure in clinical settings. Other factors, such as thrombosis, may also be associated with abnormal elevation in pressure in some cases. These factors may induce this phenomenon simultaneously. Further clinical investigations are required. Second, we did not assess the contribution of platelets to the abnormal elevation in pressure in our *ex vivo* circuit. We measured the number of erythrocytes, leukocytes, and platelets in our experiments. When in-circuit pressure was elevated, the numbers of platelets and leukocytes were decreased (Fig. 3C). Some clinical studies have also reported a decline in platelet count during CPB with or without an abnormal elevation in pressure^8,31^. Platelets are presumed to initially attach to the filter, which leads to narrowing of the space for blood to pass through. In a microfluidic study that showed shear-induced NETosis, platelets were accumulated and fluid shear stress was increased, which resulted in disruption of leukocytes^23^. Further investigations are required to understand the role of platelets in abnormal elevation in pressure in CPB.

In conclusion, our study shows that leukocyte-derived extracellular DNA contributes to abnormal in-circuit elevation of pressure in an *ex vivo* circuit. Additionally, heparin can promote the release of extracellular DNA from leukocytes.

## Methods

### Storage of porcine blood and platelet-rich plasma

All experiments were performed in accordance with the guidelines of Kagoshima University, Kagoshima, Japan. Porcine blood anticoagulated with 5 U/mL of unfractionated heparin (Mochida, Tokyo, Japan) or a 1:10 volume of 3.2% sodium citrate was purchased from Domestic Animal Resource Development Co., Ltd., Minami-kyushu, Japan. Using porcine blood anticoagulated with sodium citrate, platelet-rich plasma (PRP) was prepared by centrifugation at 400 × *g* for 15 minutes. The numbers of erythrocytes, leukocytes, and platelets were analyzed with Celltac α (Nihon Kohden Corp., Tokyo, Japan). Whole blood and PRP were stored at 4 °C for 1, 3, 5, or 7 days, and was warmed slowly back to room temperature before use.

### Preparation of the extracorporeal recirculation circuit

We developed an *ex vivo* recirculating circuit (Fig. 1A) using polyvinyl chloride tubes, a pooling reservoir (JMS Co., Ltd., Hiroshima, Japan), a roller pump (Stockert Instrument GmbH, Munich, Germany), a manometer (Migishita Seiki MFG Co., Ltd., Amagasaki, Japan), and a removable filter with the pore size of 40 µm (Merck Millipore KGaA, Darmstadt, Germany). In some experiments, a venous reservoir, an oxygenator, and an arterial line filter approved for clinical use were used instead of a removable filter (Fig. S1A). For priming, the circuit was filled with 200 mL of saline solution and the flow rate was set at 1 L/min.

### Treatment of porcine blood with acid, alkali, a low temperature, phorbol 12-miristate 13-acetate, or ethylenediaminetetraacetic acid

Lactate (Wako, Osaka, Japan) or sodium hydrate (Wako) was added to the priming solution to adjust the pH of porcine blood to approximately 6.8 or 8.0. The pH values of porcine blood were analyzed with Epoc (Epocal Inc., Ottawa, Canada). For adjusting the blood temperature, the reservoir was warmed up or cooled down with a water bath. In some experiments, porcine blood was stimulated with 0.6 µg/mL phorbol 12-miristate 13-acetate (PMA) (Wako) at 37 °C for 120 minutes before adding to the circuit. For inhibiting endogenous DNase activity, EDTA (Wako) was added to the priming solution at a concentration of 5 mM.

### Monitoring of in-circuit pressure

In each experiment, 100 mL of porcine blood was administered into the reservoir and the flow rate of blood–saline solution was kept at 1 L/min. In some experiments, 5 U/mL of unfractionated heparin was administered into the reservoir at 15 minutes, and DNase I (Sigma-Aldrich, Saint-Louis, MI, USA), RNase A (Sigma-Aldrich), or proteinase K (Sigma-Aldrich) was administered into the reservoir at 30 minutes. In-circuit pressure was monitored every 30 seconds. Experiments were terminated at 60 minutes or at the time when in-circuit pressure reached 525 mmHg, which was the safety limit of the circuit. The filter was removed at the end of the experiments and analyzed by immunofluorescent techniques. All procedures were performed at room temperature.

### Immunofluorescent analysis

The filter was fixed with OptiLyse C (Beckman Coulter, Marseille, France) for 15 minutes and then treated with phosphate-buffered saline (PBS) for 5 minutes. After three washes with PBS containing 0.1% triton (PBST), unspecific binding sites were blocked by incubation with PBST containing 1% bovine serum albumin (Sigma-Aldrich) for 60 minutes. The filter was then incubated with 60 µg/mL rabbit anti-fibrinogen antibody (Agilent Dako, Santa Clara, CA, USA) in PBST containing 1% bovine serum albumin for 60 minutes. After three washes with PBS, the filter was incubated with 20 µg/mL goat anti-rabbit antibody conjugated with Alexa Fluor 594 (Life Technologies, Eugene, OR, USA) for 60 minutes in the dark. After washing, the filter was incubated with 2 µM 4′,6-diamidino-2-phenylindole dihydrochloride (Dojindo, Kumamoto, Japan) for 5 minutes in the dark. Whole images of the filters were analyzed with the All-in-One Fluorescence microscope BZ-X700 (Keyence Corp., Osaka, Japan) and details were analyzed with the confocal microscope LSM 700 (Zeiss, Oberkochen, Germany).

### Statistical analysis

Data are shown as mean ± standard deviation (SD). For comparison between groups, the area under the in-circuit pressure curve was calculated. Repeated measures analysis of variance models were used to investigate changes in the area under the curve (AUC) over time (days). Paired t-tests were used to analyze the difference between two groups. Statistical analysis was performed with SPSS software, version 21.0 (IBM, Inc., Chicago, IL, USA). A p value of <0.05 was considered significant.

## Supplementary information

Supplementary Figures.

Video S1.

Video S2.
