# Supplementary material for: Leukocyte-derived extracellular DNA contributes to abnormal pressure elevation in the extracorporeal circulation circuit
Source: Sci Rep. 2020 Jan 16;10:474. doi: 10.1038/s41598-019-57173-5 (PMC6965310; doi:10.1038/s41598-019-57173-5)
Supplement: Supplementary file 1 — Supplementary Figures. [file 41598_2019_57173_MOESM1_ESM.pdf]

Leukocyte-derived extracellular DNA contributes to abnormal pressure elevation in the extracorporeal circulation circuit

Nozomi Yashima,[1-3] Takashi Ito,[1-2] Kenji Kajiyama,[4] Hiroyuki Maeda,[4] Yasuyuki Kakihana,[1] and Ikuro Maruyama[2]

[1]Department of Emergency and Intensive Care Medicine, Kagoshima University Graduate School of Medical and Dental Sciences, Kagoshima, Japan; [2]Department of Systems Biology in Thromboregulation, Kagoshima University Graduate School of Medical and Dental Sciences, Kagoshima, Japan; [3]Department of Anesthesiology, Yamagata University Graduate School of Medical Science, Yamagata, Japan; [4]Cardiovascular Device Team, Development Department, Surgical & Therapeutical Business Unit, JMS Co., Ltd., Hiroshima, Japan

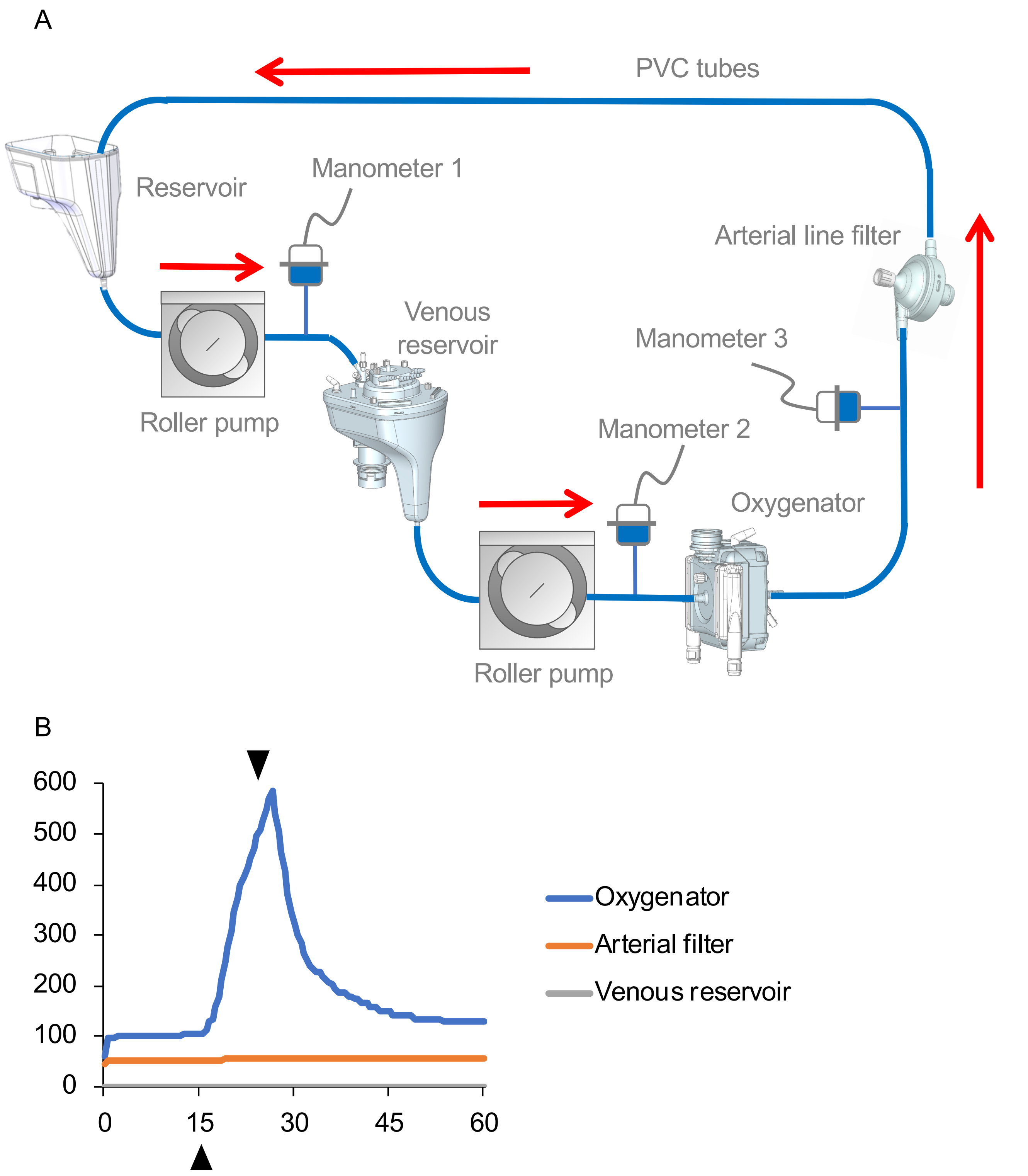

Supplementary Fig. S1. An abnormal elevation in pressure occurred in the circuit using clinically approved devices. (A) A schema of the extracorporeal recirculation circuit using the clinically approved devices is shown. (B) A representative in-circuit pressure was shown. In-circuit pressure was elevated at the oxygenator after adding heparin (▲). Furthermore, DNase treatment (▼) immediately decreased in-circuit pressure. Porcine blood stored for 7 days was used.

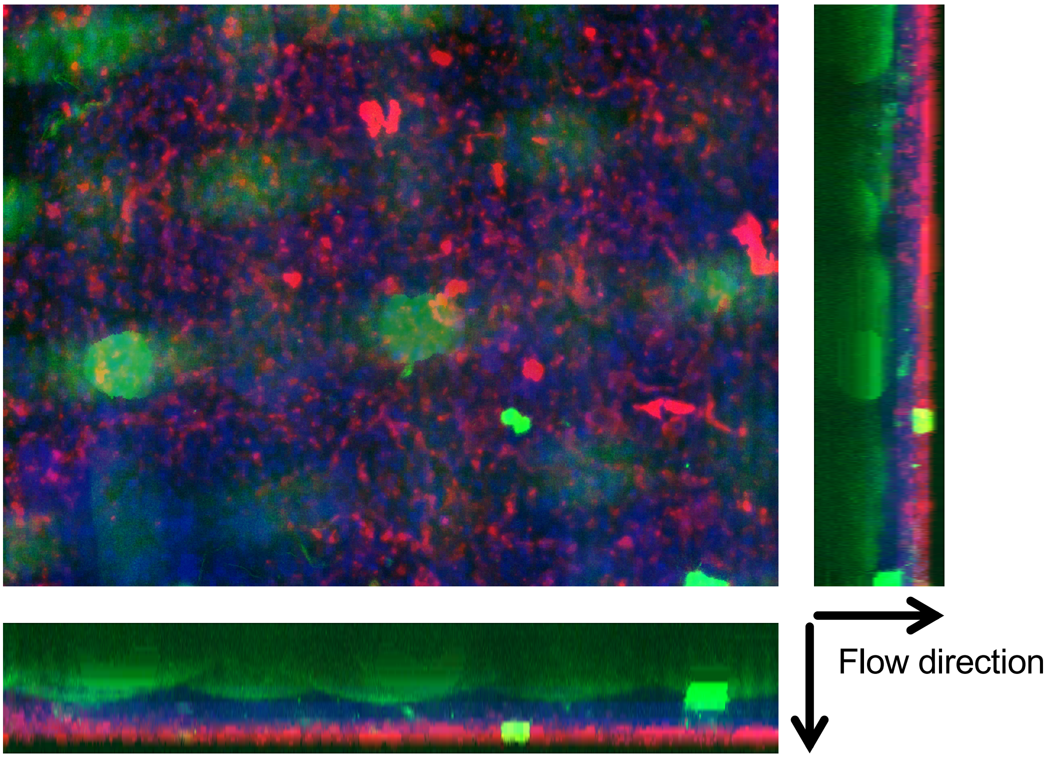

Supplementary Fig. S2. The relative position of the filter, DNA, and fibrin(ogen). A three-dimensional reconstitution technique showed that the extracellular DNA layer was located immediately downstream of the filter. The fibrin(ogen) layer was located downstream of the DNA layer. The filter, DNA, and fibrin(ogen) are labeled in green, blue, and red, respectively.

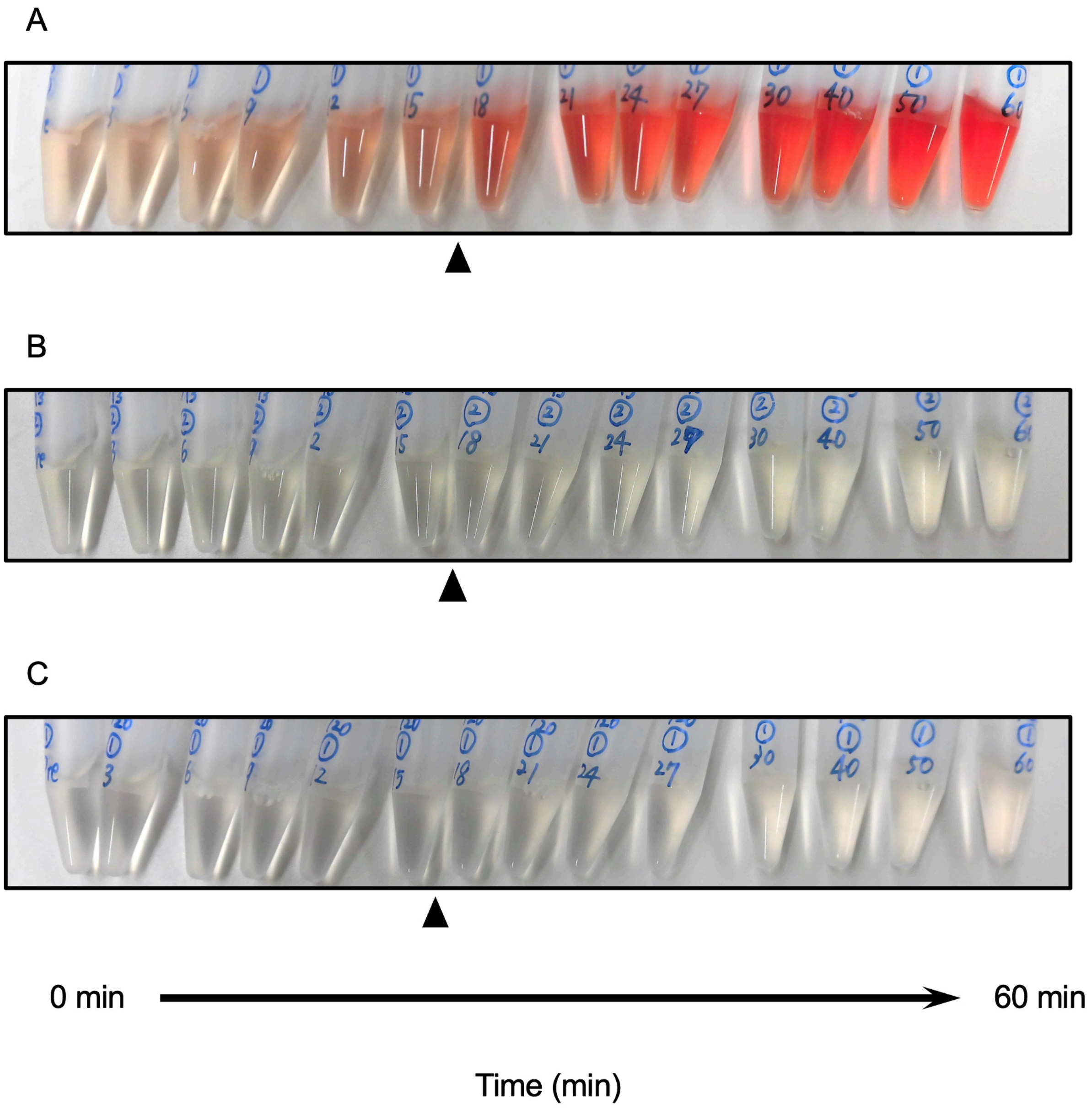

Supplementary Fig. S3. Hemolysis is observed in associated with elevation of in-circuit pressure. (A) Plasma samples were assessed over time while whole blood (day 7) passed through the filter. (B) Plasma samples were assessed over time while PRP (day 7) passed through the filter. (C) Plasma samples were assessed over time while whole blood (day 0) passed through the filter. Arrow heads indicate the timing of heparin administration in the circuit.

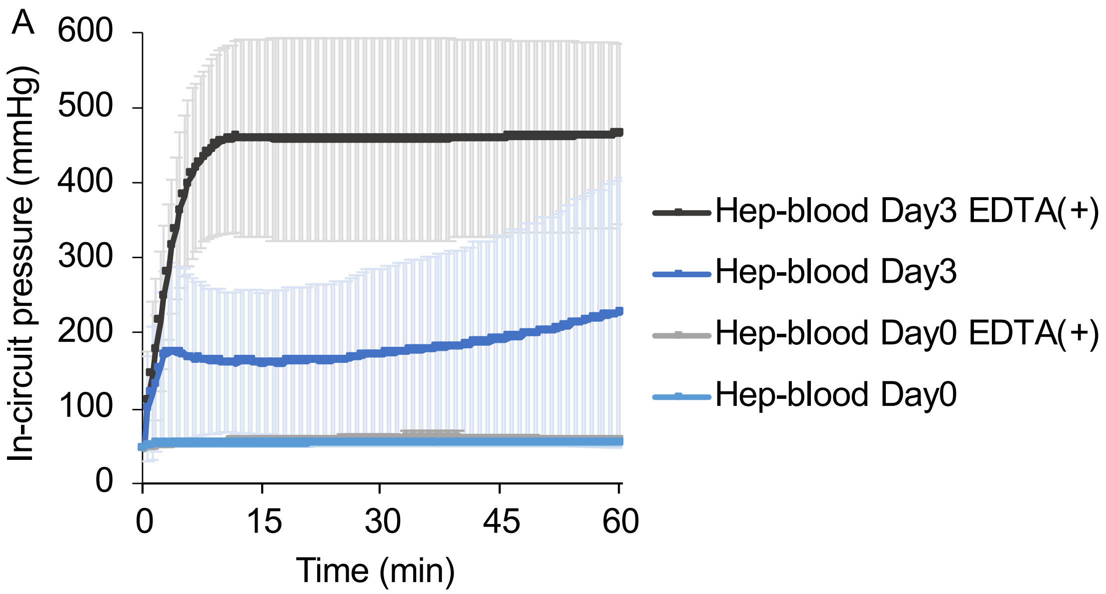

Supplementary Fig. S4. Inhibition of endogenous DNase by EDTA increases in-circuit pressure. Inhibition of endogenous DNase by EDTA did not increase in-circuit pressure when using heparinized blood on day 0 (EDTA positive vs negative: 57.2 ± 4.7 mmHg vs 55.3 ± 5.6 mmHg at 60 minutes), but significantly increased in-circuit pressure when using heparinized blood on day 3 (EDTA positive vs negative: 465.0 ± 120.0 mmHg vs 226.9 ± 179.8 mmHg at 60 minutes, $p < 0.05$).

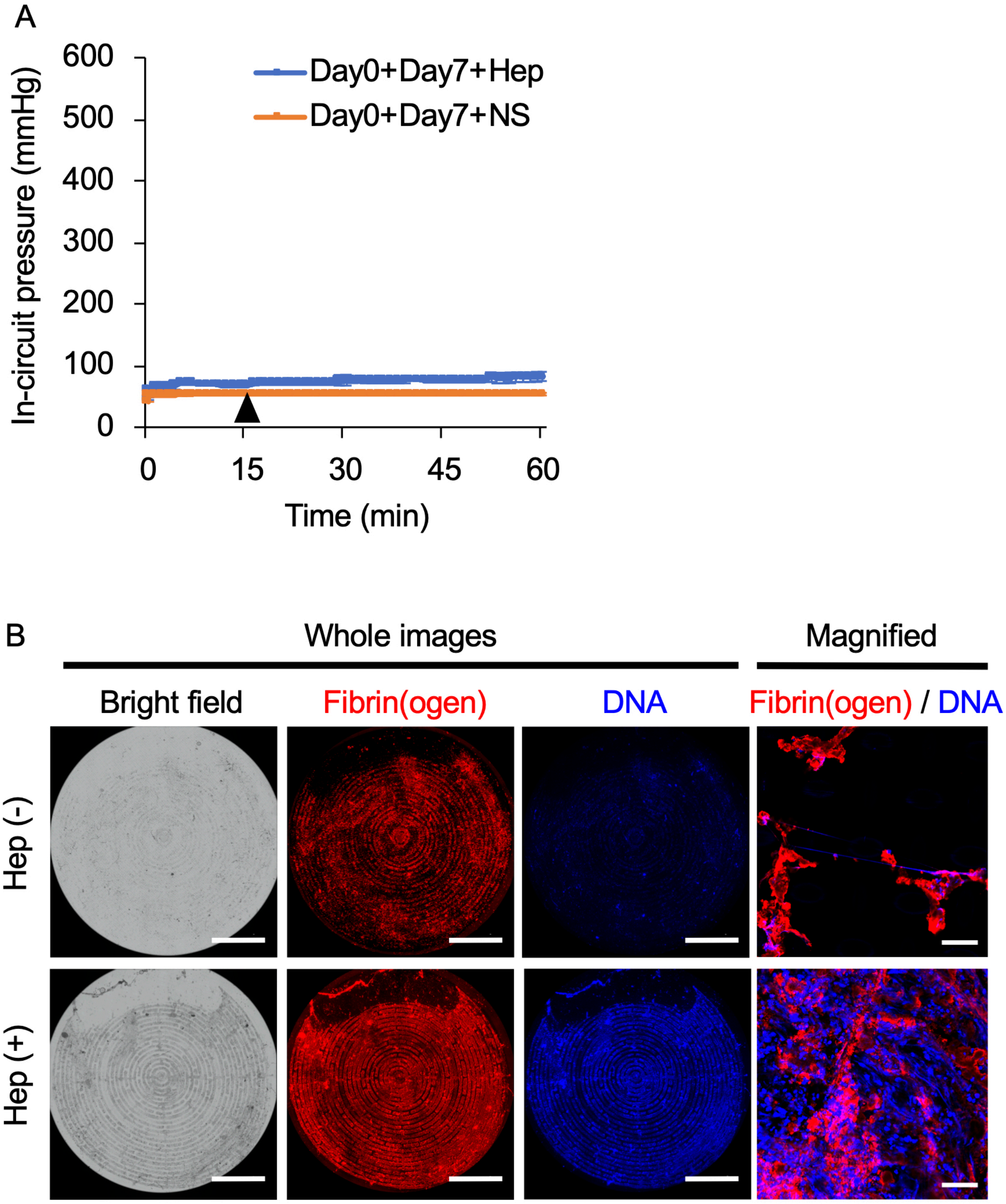

Supplementary Fig. S5. Heparin administration into the fresh/old blood mixture leads to extracellular DNA deposition. (A) Unfractionated heparin (5 U/mL) was administered into the mixture of saline (200 mL), fresh porcine blood (300 mL), and 7-day-old porcine blood (100 mL) at 15 minutes. In-circuit pressure was monitored for 60 minutes. N = 3 per group. (B) The filter was removed at 60 minutes and analyzed by immunofluorescence. Fibrin(ogen) was labeled in red and DNA was labeled in blue. Representative images of the whole filter (scale bars: 10 mm) and the magnified view (scale bars: 50 μm) are shown.

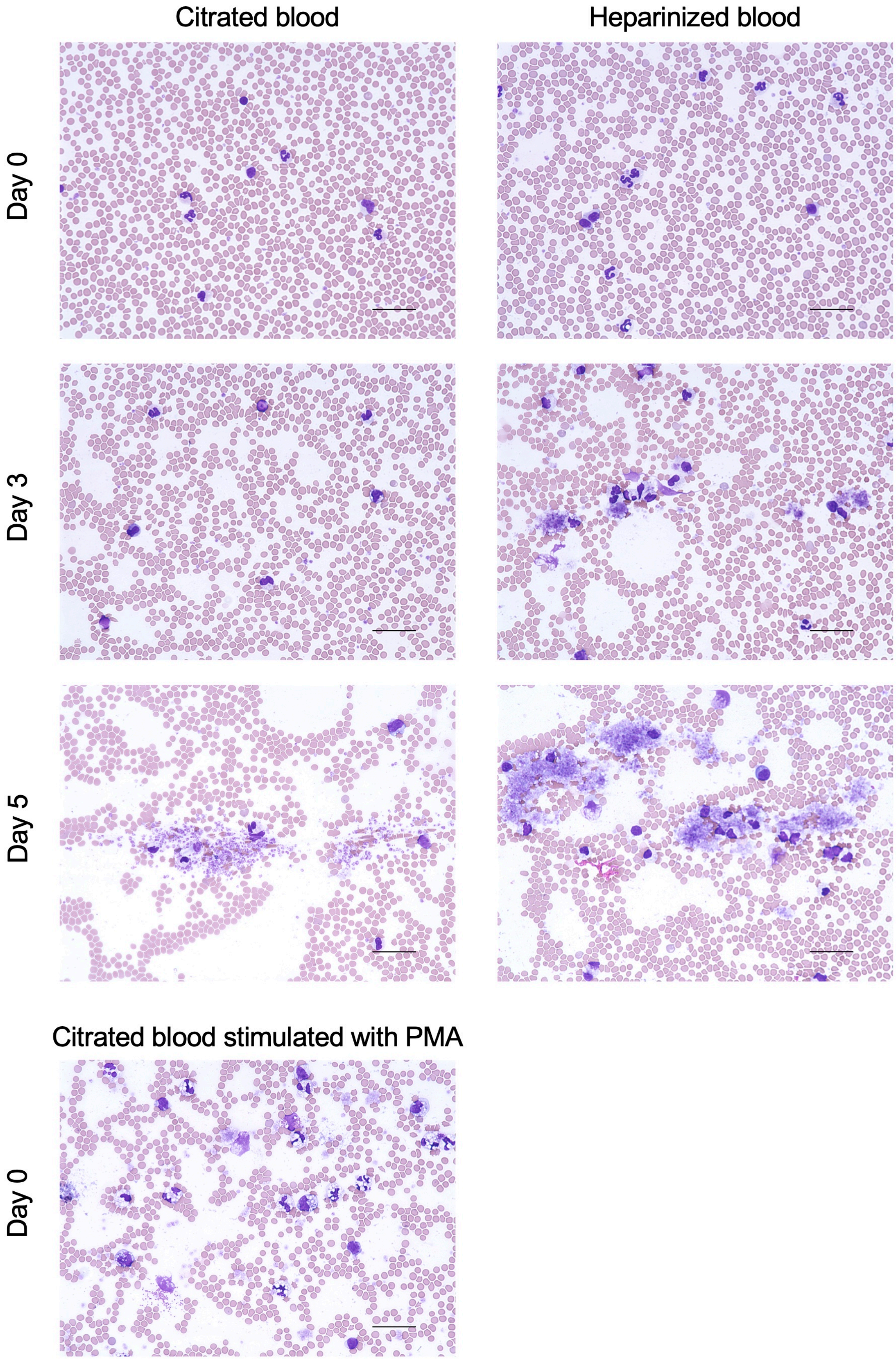

Supplementary Fig. S6. Giemsa-stained blood smears. In heparinized blood, leukocytes were aggregated with platelets earlier and in a greater amount compared with citrated blood. PMA-stimulated leukocytes showed nuclear degeneration and cytoplasmic vacuolization, and many of these leukocytes were disrupted. Scale bars: 40 μm.
